# The impact of dietary diversity and seasonality in food availability on the quantile distribution of birth size among pregnant women in rural Malawi – a cross-sectional study

**DOI:** 10.1186/s12884-022-04924-4

**Published:** 2022-07-27

**Authors:** Katrine G. Hjertholm, Gerd Holmboe-Ottesen, Ibrahimu Mdala, Zumin Shi, Per O. Iversen

**Affiliations:** 1 Department of Nutrition, IMB, University of Oslo, P.O. Box 1046 Blindern, 0317 Oslo, Norway; 2 Department of Community Medicine and Global Health, Institute of Health and Society, University of Oslo, Oslo, Norway; 3 Human Nutrition Department, College of Health Sciences, QU Health, Qatar University, Doha, Qatar; 4 Department of Haematology, Oslo University Hospital, Oslo, Norway; 5 Division of Human Nutrition, Stellenbosch University, Tygerberg, South Africa

**Keywords:** Dietary diversity score, Neonatal anthropometry, Seasonality, Malawi, Pregnancy, Quantile regression

## Abstract

**Background:**

Dietary diversity scores can be used as a proxy for dietary intakes and for assessment of nutrient adequacy. Studies from low-resource settings have found maternal dietary diversity scores to be associated with neonatal birth size. We here investigated the relationship between the dietary diversity score among pregnant mothers and birth size of their offspring across quantiles of the birth size variables; birth weight, length, abdominal circumference, and head circumference. We also investigated if seasonality affects birth size across different quantiles.

**Methods:**

Dietary intake and anthropometric data were collected from 190 pregnant women and their neonates in rural Malawi through two agricultural seasons. Dietary data was collected using 24-hour recall interviews and was categorized into the 10-food group dietary diversity score proposed for women by the Food and Agriculture Organization. Neonatal anthropometrics were collected upon delivery at health facilities. Quantile regression analyses were used to investigate associations between dietary diversity scores and birth size, as well as between seasonality and birth size.

**Results:**

We found that neonatal abdominal circumference was 0.9 cm larger during the post-harvest season compared to the pre-harvest season among neonates in the 25th quantile. Birth weight was 281.4 g higher for those born during the post-harvest season in the 90th quantile. For a one-unit increase in maternal dietary diversity score, birth weight increased by 56.7 g among those in the 25th quantile and neonatal head circumference increased by 0.2 cm for those in the 70th quantile. However, these findings did not remain significant when considering the cluster effect of the neonatal anthropometric data.

**Conclusions:**

Our findings indicate that the relationship between seasonality and birth size differs across the distribution of birth size. Investigating the effect of seasonality across the distribution of birth size could be important to identify vulnerable subgroups and develop better, targeted interventions to improve maternal and child nutrition and health.

**Supplementary Information:**

The online version contains supplementary material available at 10.1186/s12884-022-04924-4.

## Background

Dietary diversity score (DDS), which is the count of food groups consumed over a defined time period, can be used as a simple tool for measuring diet adequacy [1]. The minimum dietary diversity score for women (MDD-W) based on 10 food groups has been developed by the Food and Agriculture Organization of the United Nations (FAO) as a standard proxy indicator for nutrient adequacy [2]. A DDS of ≥5 out of 10 food groups is set as a cut-off point for acceptable nutrient adequacy [2]. Studies in low-income countries have found associations between DDS and birth size, and studies including a recent systematic review, concluded that low DDS is associated with increased risk of low birth weight newborns [3, 4].

Previous studies have usually analyzed associations between DDS and birth outcomes assuming a constant linear relationship between DDS and birth outcomes, and thus only the average effect of DDS on birth outcomes has been examined. However, when conducting interventions to improve diet and birth outcomes it is important to know the full extent of the relationship between the studied variables. Thus more informatively, one can investigate whether the relationships between DDS and the birth size variables change across the entire distribution of the birth size variables [5]. This could thus help identify vulnerable subgroups, which is important when implementing targeted interventions to improve maternal and child interventions. A study from urban South Africa found that dietary patterns had differential effect on quantiles of birth weight [6], but to our knowledge, the effect of DDS on different quantiles of birth size has not been studied.

In farming communities in low-income countries, the seasonal changes in food availability and access, such as those caused by agricultural production cycles and variation in household income, may affect the adequacy of maternal dietary intake [7]. Seasonal changes in maternal dietary intake may in turn affect neonatal birth size [8]. For example, in rural India, babies born from mothers who experienced the whole pregnancy in the food plenty season were on average 90 g heavier than babies who had most of their gestational growth in the food poor season [8]. Nutrient-restrictions during food-poor seasons may lead to epigenetic changes important for fetal growth [9].

Using the standard DDS with ten food groups suggested by FAO, as a proxy for maternal dietary intake, we here aimed to investigate the effect of maternal DDS on the distributions of birth weight, length, abdominal circumference and head circumference. Moreover, the association between MDD-W (a dichotomous variable) and the same four indicators for birth size was determined. We also wanted to examine whether there is a seasonal effect on birth size in the Malawian setting, and whether it differs across the distribution of birth size.

## Methods

### Study area and participants

A cross-sectional study was conducted in the Nankumba Traditional Authority (TA) of Mangochi District. This is a rural area with a population of about 150,000 at the time of the study. The most common occupations are subsistence farming and fishing. There is only one rainy season occurring from November to May [10, 11]. The most important staple food cultivated is maize, which is normally harvested in April–May. Other important crops are sweet potato, cassava and rice. The crops are often too small to last the whole year and the farming lacks diversification. Crop failure and increased food prices, often due to weather shocks, may thus have detrimental effects on the household food security [12]. Five health centers and one community hospital provide free health services, including antenatal- and delivery care, to the local community. The quality of antenatal care varies between centers and among visits.

Pregnant women were selected into the survey using a one-stage randomized sampling procedure. Initially, 76 clusters, defined by geographic areas (villages) were randomly selected. Only women between 28 and 35 weeks of gestation were recruited because we wanted to study participants in their last trimester, but who would not give birth before the 10-days required for data collection. Gestational age was derived from last menstruation cycle or through assessment of fundal length. Women who were bedridden due to illness or had not been residents in the study area for the previous 6 months, were excluded. From each cluster, 4–6 participants were recruited. To avoid selection bias in the recruitment, all potential participants in the geographical cluster were identified by local health volunteers or health surveillance assistants. In most cases, all the eligible pregnant women within a cluster were invited to participate.

Results from this paper are part of a cross-sectional pre-study study aiming to inform a planned food-based intervention trial to improve maternal diet and birth size in the study area. The sample size was calculated to detect the prevalence of iron deficiency of 26% to within ±10%, with a power of 80%, at a 95% confidence level, assuming a design effect of 1.5 and a 35% rate of attrition. A design effect of 1.5 was added to account for similarities between participants recruited from the same geographic clusters [13]. The calculations were executed using an online sample size calculator [14].

To sensitize the local community to the project, chiefs from all villages of the Nankumba TA were invited to an information meeting held by the study coordinator. Soap was given as incentives after each interview, and participants received a bag of sugar after the last interview. Money to cover transportation to the health clinic was also provided.

Dietary data were collected from pregnant women between August and September 2013, when food availability was sufficient for most households, and between February and March 2014, when food was limited. Some participants gave birth in another agricultural season than the dietary interview took place. Participants were classified according to post-harvest (food plenty) and pre-harvest season (food poor) dependent on the season in which they gave birth to assess seasonality of birth outcomes. Infant weight, length, head circumference and abdominal circumference were measured at birth. All data were collected by trained Malawian interviewers with a university degree in nutrition or agriculture who were fluent in both English and the local language Chichewa.

### Dietary data

During a 10-day period, we collected multiple quantified and semi-quantified dietary recall data from the participants in their own homes in order to encourage participation and improve recall of the foods consumed [15]. More comprehensive descriptions of dietary intakes have previously been published [16, 17]. For the present purpose, only one 24-hour recall was used to investigate if DDS could be a simple proxy tool for dietary intake, taking into account that multiple 24-hour recalls are often too resource-demanding in such settings. A reference period of 24 hours (compiled from the first day of dietary interviews) was therefore chosen to investigate associations between DDS and birth size. Detailed description of the interactive multiple pass 24-hour recall method used has previously been described [16]. To enhance memory, the participants were asked to prospectively mark on pictorial charts all foods and beverages consumed during the day.

DDS is defined as the number food groups consumed over a reference period and used as a qualitative proxy for an individual’s dietary adequacy [18]. The food items included were based on 10 food groups as recommended by the FAO and the USAID FANTA project: (i) grains and white roots and tubers, (ii) pulses, (iii) nuts and seeds, (iv) dairy, (v) meat, poultry and fish, (vi) eggs, (vii) dark green leafy vegetables, (viii) other vitamin A-rich fruits and vegetables, (ix) other vegetables, (x) other fruits. No minimal amount was required for a food item to be included. Each food group was weighted equally, with the value of 1.

### Sociodemographic data

A pre-coded questionnaire developed and used in rural Malawi, was administered at the first visit to collect socio-demographic data on both participant and household level, such as age, education (i.e. completed at least primary school), occupation, number of children, number of people in the household, and food security. As a proxy for household economic status a household asset index was calculated based on 11 household items given scores according to their monetary value [19].

### Neonatal anthropometry

Anthropometric measurements of the newborn infants were collected at health facilities by trained nurses within one hour after birth. Birth weight (nude) was measured to the nearest gram using Seca 376 digital pediatric scales (Hamburg, Germany). Length was measured to the nearest 0.1 cm using Seca 233 infantometer. Both head and abdominal circumference were measured to the nearest 0.1 cm using Seca 212 non-stretchable measuring tape. All neonatal anthropometric measurements were collected according to the International Fetal and Newborn Growth Standards for the twenty-first Century and World Health Organization standard procedures [20, 21].

### Statistical methods

We applied independent t-test and test of proportions (chi-square test of association) to compare background characteristics between participants with and without neonatal data, and to compare background characteristics between participants in the two seasons. Mean birth weight, length, head circumference and abdominal circumference are presented for each season, and differences between seasons were compared using independent t tests. To visualize distributions of birth outcomes by birth seasons, Kernel density curves were made using kdensity syntax in Stata. Barnard’s exact test was applied to compare proportions of low birth weight between the two seasons.

Quantile regression, as introduced by Koenker and Basset [5], was used instead of ordinary least square regression to provide a more comprehensive view of the effect of the independent variable on the dependent variable across the distribution of the dependent variable. Quantile regression was used to estimate associations between DDS and changes in birth outcomes at the 25th, 50th, 75th and 90th quantiles, adjusted for covariates (maternal age, household socioeconomic status, education, birth season, interview season, total duration of pre-harvest season during pregnancy and maternal energy intake). The adjustment of covariates was based on the current knowledge of the determinants of birth outcome. Some variables (e.g. season, duration of pre-harvest season) were included as we believe they may represent some unmeasured confounding factors. The analyses were conducted using both sqreg and qreg2 commands in Stata. Sqreg was applied to provide the quantile regression coefficient plots. Only quantile regression coefficient plots of statistically significant data are presented. Data on neonatal anthropometry were clustered within geographical areas, thus giving reason to assume that the anthropometry was correlated within these areas. We therefore also applied qreg2 to adjust for the cluster effect. Effect estimates are presented as changes in grams for birth weight, centimeters for length, head circumference and abdominal circumference when DDS increases by one unit. Quantile regression was also applied to investigate differences between birth season (the pre-harvest vs post-harvest) and birth outcomes at the 25th, 50th, 75th and 90th quantile, using both sqreg and qreg2. Effect estimates are presented as differences in grams for birth weight and centimeters for length, head circumference and abdominal circumference between the pre-harvest and post-harvest season. All analyses were performed in Stata/SE 16.0. Statistical significance was set at *P* < 0.05.

## Results

### Participant characteristics

**Fig. 1:**
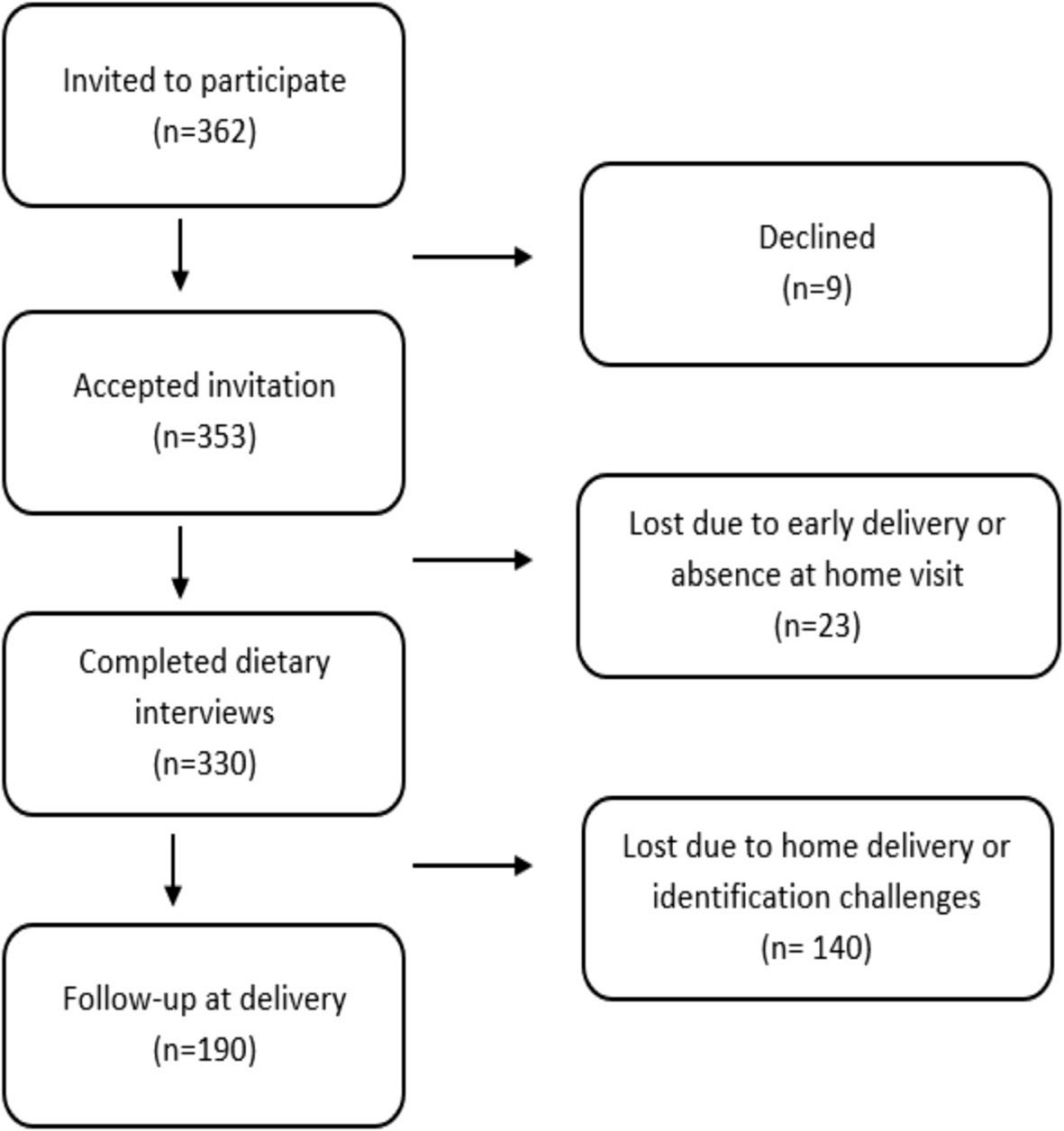
Flow chart of the inclusion process. Of the 362 participants invited into the study, 353 accepted. Of these, 330 completed all dietary interviews and 190 were followed up at the health facility upon delivery

In total 362 participants were invited to participate (Fig. 1). Of these, 353 accepted and 330 completed all dietary interviews. We collected data from 190 (56.6%) of these women at the health clinics upon delivery. There were no statistical differences between those with or without neonatal anthropometric data. Among those with anthropometric data, 136 (71.6%) gave birth during the post-harvest season and 54 (28.4%) during the pre-harvest season. There were no significant differences in sample characteristics between participants who gave birth during post- and pre-harvest seasons (Table 1).

**Table 1:** Maternal characteristics and neonatal size

	Post-harvest	Pre-harvest	Total	
	(*n* = 136)	(*n* = 54)	(n = 190)	*P*-value
**Age of participant**				0.66
Mean (SD) years	24.9 (6.5)	25.3 (6.9)	25.0 (6.6)	
**Household assets**				0.78
Median (quartiles 1, 3)	4.1 (2.1, 7.0)	3.5 (1.5, 5.3)	3.9 (1.8, 6.8)	
**Number of previous births**				0.88
0	34 (25.0%)	15 (27.8%)	83 (25.2%)	
1–2	50 (36.8%)	18 (33.3%)	115 (35.0%)	
> =3	52 (38.2%)	21 (38.9%)	131 (39.8%)	
**Education**				0.23
No	42 (30.9%)	12 (22.2%)	93 (28.3%)	
Yes	94 (69.1%)	42 (77.8%)	236 (71.7%)	
**Neonatal anthropometry**				
**Birth weight**				0.25
Mean (SD) grams	3111.4 (380.0)	3181.7 (381.6)	3131.5 (380.8)	
**Birth length**				0.31
Mean (SD) cm	48.2 (2.8)	48.6 (1.9)	48.3 (2.6)	
**Head circumference**				0.40
Mean (SD) cm	34.7 (1.7)	34.9 (1.3)	34.8 (1.6)	
**Abdominal circumference**				0.51
Mean (SD) cm	30.6 (2.7)	30.9 (1.5)	30.7 (2.4)	

*SD* standard deviation

### Neonatal anthropometry

**Fig. 2:**
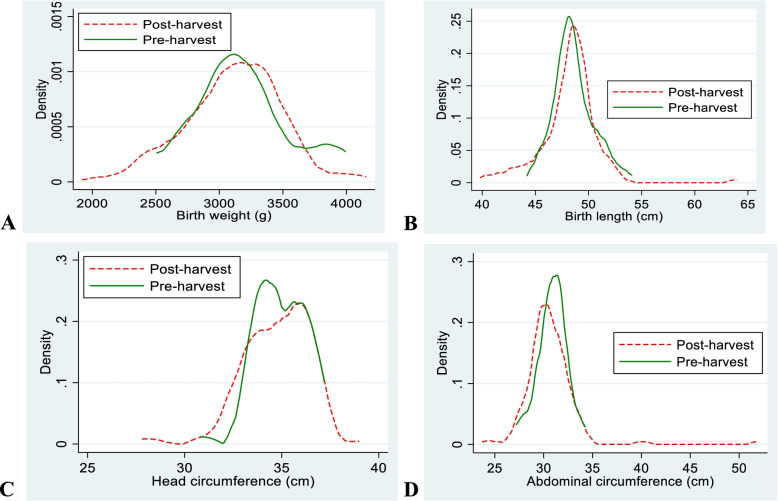
Kernel density curves showing the distribution of birth weight (**A**), birth length (**B**), head circumference (**C**) and abdominal circumference (**D**) by birth season (post-harvest and pre-harvest season). For birth weight and birth length the data distributions are slightly skewed to the right for the post-harvest season

The distributions of birth size by birth seasons are shown as Kernel density curves (Fig. 2). There was a slight right-skewed distribution of birth weight and birth length in the post-harvest season compared to the pre-harvest season, indicating larger birth size. However, using independent t-test, no statistically significant differences were found in mean birth weight, length, abdominal circumference or head circumference between the two seasons (Table 1). However, when using quantile regression based on qreg2 command in Stata, we found that in the 90th quantile of birth weight, infants born during the post-harvest season were 350 g heavier than those born in the pre-harvest season in the unadjusted model and 281 g heavier in the adjusted model (Table 2). In both unadjusted and adjusted models, newborns in the 25th quantile of abdominal circumference had a larger abdominal circumference when born during the post-harvest season. Furthermore, in the adjusted model, the abdominal circumference was 0.9 cm larger in the post-harvest season compared to the pre-harvest season, among those in the 25th quantile. We obtained rather similar results using the sqreg command in Stata (Table S1, Additional file 1). The prevalence of low birth weight was 5.9% (*n* = 8) in the post-harvest season, whereas none of the infants in the pre-harvest season were born with low birth weight (*P* > 0.05).

**Table 2:** Associations between birth seasons (pre-harvest vs post-harvest) and birth size

	25th quantile (95% CI)	50th quantile (95% CI)	70th quantile (95% CI)	90th quantile (95% CI)
**Birth weight**				
Unadjusted	10	−25.0	−14.0	350.0**
	(− 198.1, 218.1)	(− 195.3, 145.3)	(− 179.1, 151.1)	(131.1, 568.9)
Adjusted	−89.9	−82.9	− 149.1	281.4*
	(− 302.8, 123.1)	(− 327.5, 161.6)	(− 322.4, 24.2)	(25.5, 537.4)
**Birth length**				
Unadjusted	0.3	−0.2	− 0.2	0.5
	(−0.6, 1.2)	(−0.9, 0.5)	(−1.2, 0.8)	(− 0.7, 1.7)
Adjusted	−0.1	− 0.4	− 0.6	0.0
	(−1.1, 1.0)	(−1.3, 0.4)	(− 1.5, 0.3)	(−2.3, 2.2)
**Head circumference**				
Unadjusted	0.3	0.1	0	−0.1
	(− 0.4, 1.0)	(− 0.6, 0.8)	(− 0.6, 0.6)	(− 0.5, 0.3)
Adjusted	− 0.1	− 0.2	− 0.4	0.0
	(−1.5, 1.3)	(−1.1, 0.6)	(− 1.2, 0.3)	(− 0.7, 0.6)
**Abdominal circumference**				
Unadjusted	0.6	0.4	0.3	−0.1
	(− 0.2, 1.4)	(− 0.3, 1.1)	(− 0.2, 0.8)	(− 0.9, 0.7)
Adjusted	0.9.**	0.7	0.8	0.6
	(0.2, 1.6)	(0.0, 1.3)	(−0.1, 1.7)	(−1.1, 2.3)

Data is presented as quantile regression estimates and 95% confidence intervals (CI) of birth seasons for the 25th, 50th, 70th and 90th quantiles of birth outcomes (*n* = 190). Adjusted for maternal age, household assets, maternal education (yes vs no), interview season, total duration of pre-harvest season during pregnancy and maternal energy intake. *P < 0.05, **P < 0.01

### Associations between maternal DDS and neonatal anthropometry

**Fig. 3:**
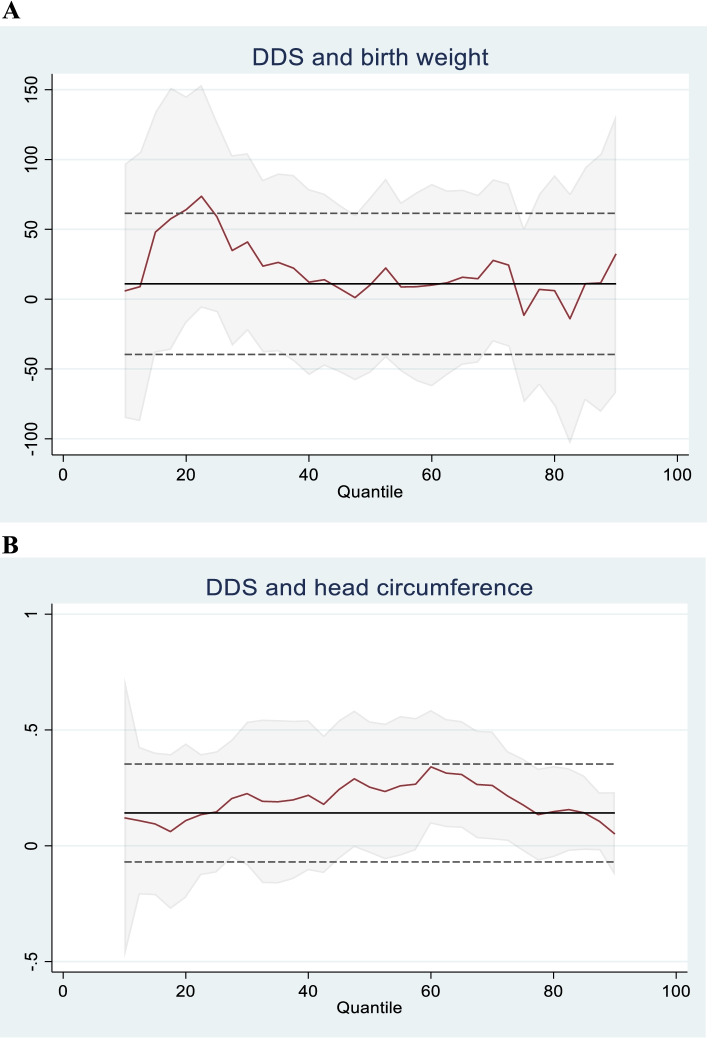
Graphical illustrations of quantile regression coefficients (red line) with the corresponding 95% CI (shaded area) for DDS associated birth weight (**A**) and head circumference (**B**)

We found that DDS was positively associated with birth weight in the 25th quantile, when adjusted for covariates (Table 3): A one-unit increase in DDS was associated with 56.7 g increase in birth weight. No statistically significant associations were found between DDS and birth weight in the other quantiles. For head circumference, a one-unit increase in DDS was associated with a 0.2 cm increase among those in the 70th quantile. Graphical illustrations in Fig. 3 show how the effect of DDS on head circumference and birth weight varied across quantiles. When applying qreg2 to adjust for the cluster effect, the regression coefficients and confidence intervals remained almost identical, but no associations were statistically significant in the adjusted models (Table 4). Moreover, we found no significant associations between MDD-W, as a dichotomous variable, and birth outcomes (data not shown).

**Table 3:** Association between dietary diversity score and birth size

	25th quantile (95% CI)	50th quantile (95% CI)	70th quantile (95% CI)	90th quantile (95% CI)
**Birth weight**				
Unadjusted	17.5	−5.0	26.3	31.0
	(− 65.1–100.1)	(− 55.6–45.6)	(−48.2–100.7)	(−59.8–121.8)
Adjusted	56.7*	30.6	8.3	32.0
	(3.7–109.6)	(−38.3–99.4)	(−63.8–80.4)	(− 102.2–166.1)
**Birth length**				
Unadjusted	0.1	0	0	− 0.2
	(− 0.4–0.6)	(− 0.2–0.2)	(−0.3–0.3)	(−0.6–0.2)
Adjusted	0.2	0.2	0.2	−0.2
	(−0.3–0.7)	(− 0.1–0.5)	(0.0–0.5)	(− 0.5–0.2)
**Head circumference**				
Unadjusted	0.1	0.2	0.1	0.0
	(−0.1–0.3)	(− 0.3–0.6)	(− 0.2–0.3)	(−0.1–0.1)
Adjusted	0.1	0.2	0.2*	0.0
	(−0.2–0.5)	(0.0–0.5)	(0.0–0.5)	(−0.3–0.4)
**Abdominal circumference**				
Unadjusted	−0.1	0.2	0.1	0.2
	(−0.4–0.2)	(−0.3–0.7)	(−0.2–0.5)	(−0.1–0.6)
Adjusted	0.0	0.1	0.1	0.3
	(−0.4–0.4)	(−0.2–0.3)	(−0.1–0.3)	(−0.2–0.7)

Data is presented as quantile regression estimates and 95% confidence intervals (CI) of dietary diversity scores for the 25th, 50th, 70th and 90th quantiles of birth outcomes (*n* = 190). Data are adjusted for maternal age, household assets, maternal education (yes vs no), birth season, interview season, total duration of pre-harvest season during pregnancy and maternal energy intake. *P < 0.05

**Table 4:** Association between dietary diversity score and birth size

	25th quantile (95% CI)	50th quantile (95% CI)	70th quantile (95% CI)	90th quantile (95% CI)
**Birth weight**				
Unadjusted	17.5 (−50.3, 85.3)	−5.0 (− 48.8, 38.8)	26.3 (− 18.7, 71.2)	31.0 (− 50.6, 112.6)
Adjusted	56.6 (−16.1, 129.4)	30.6 (− 23.9, 85.1)	8.3 (− 44.5, 61.1)	32.0 (− 63.0, 126.9)
**Birth length**				
Unadjusted	0.1 (− 0.2, 0.4)	0.0 (− 0.3, 0.3)	0.0 (− 0.4, 0.4)	−0.2 (− 0.4, 0.01)
Adjusted	0.2 (− 0.4, 0.8)	0.2 (− 0.2, 0.5)	0.2 (− 0.1, 0.6)	−0.2 (− 0.5, 0.2)
**Head circumference**				
Unadjusted	0.1 (−0.2, 0.4)	0.2 (−0.2, 0.5)	0.1 (− 0.1, 0.3)	0.0 (− 0.2, 0.2)
Adjusted	0.1 (− 0.2, 0.4)	0.2 (− 0.1, 0.5)	0.2 (− 0.02, 0.5)	0.05 (− 0.1, 0.2)
**Abdominal circumference**				
Unadjusted	− 0.1 (− 0.4, 0.2)	0.2 (− 0.1, 0.5)	0.1 (− 0.2, 0.4)	0.2 (− 0.1, 0.5)
Adjusted	0.1 (− 0.4, 0.3)	0.1 (− 0.2, 0.4)	0.1 (−0.1, 0.3)	0.3 (− 0.2, 0.7)

Data is presented as quantile regression estimates and 95% confidence intervals (CI) of dietary diversity scores for the 25th, 50th, 70th and 90th quantiles of birth outcomes (n = 190). Data are adjusted for maternal age, household assets, maternal education (yes vs no), birth season, interview season, total duration of pre-harvest season during pregnancy and maternal energy intake. The clustering effect of neonatal anthropometry is also considered in this analysis

## Discussion

In a rural Malawian setting, we investigated the effects of seasonality on birth size (measured by birth weight, birth length, abdominal circumference and head circumference), and the potential associations between DDS and the same four indicators of birth size using quantile regression.

In adjusted analyses, we found that babies in the 90th quantile who were born during the post-harvest season were 281 g heavier than those born during the pre-harvest season. Our results support previous findings, e.g. from the Pune Maternal Nutrition Study where babies exposed to the season with greatest food availability in late gestation were heavier than those exposed to food lean season in late gestation [8]. However, our findings suggest only seasonal differences in the 90th quantile, indicating that smaller babies were not affected by seasonal variation. A previous study from Malawi found that the association between season and birth weight was only moderate, and it was influenced by the year of study, implying that such findings should be interpreted with caution as results could differ from one year to another [22]. We found a significant association between season of birth and abdominal circumference in the 25th quantile both in unadjusted and adjusted analyzes. The abdominal circumference was 0.9 cm larger during post-harvest season compared to pre-harvest season among the neonates in the 25th quantile. Lower abdominal circumference in the pre-harvest season could be due to reduced fetal growth and visceral size e.g. resulting from maternal malnutrition [23]. However, the effect size is quite small and due to a large confidence interval this result was likely to be weakened by a low precision estimate. As seasonality could play a role in neonatal size in this setting, it would be useful to investigate further the underlying causes of these differences and take this into account when developing interventions to improve birth size. As we did not detect associations between DDS and birth size in the fully adjusted models, the seasonal effect on birth size could be mediated through e.g. increased work load or prevalence of infections during the pre-harvest season, rather than changes in dietary intakes. However, taking into account the below mentioned limitations in this study, we cannot rule out that dietary intake, here measured by DDS, in fact is a mediator for seasonal changes in birth size in this setting.

The national prevalence of low birth weight in Malawi around the time of our study was 12% [10]. We found a prevalence of only 5.6% in the post-harvest season and no low birth weight infants in the pre-harvest season. These discrepancies might be due to an insufficient sample size to measure the prevalence accurately. Moreover, women with complicated births and preterm deliveries, or histories of complications were often transferred to a larger hospital and therefore not captured at the health facilities included in our study. Furthermore, even if delivering at one of the study facilities, if births were complicated, often in cases of low birth weight, measurements for the study might not have been prioritized.

Examining associations between dietary diversity and birth size is important when developing nutrition education interventions, as dietary advice is given on food level, and not on single nutrients. Few studies have examined DDS in association with other birth outcomes than birth weight. Moreover, to our knowledge, quantile regression has not been applied when investigating the relationship between DDS and birth outcomes among rural pregnant women. We did not find any associations between DDS and birth outcomes when adjusting for covariates, including the geographical clustering effect of neonatal anthropometry, nor between MDD-W as a dichotomous variable and birth outcomes. Several studies have found that dietary diversity is positively associated with birth weight and with reduced risk of low birth weight [3, 4, 24, 25]. Our findings are not easily compared to other studies due to differences in analytical methods. However, a study from South Africa found that dietary patterns affected birth weight in different quantiles using quantile regression [6]. Based on previous findings from others, our results are somewhat unexpected. Several issues could explain this. Firstly, the small sample size in this study increase the risk of type II errors, i.e. producing false negative results. To further investigate the differential effects of DDS on birth size, a similar study with a larger sample size should be conducted. Secondly, based on previous findings from this project, perhaps certain food groups are better predictors of birth size than the overall DDS. We have reported that frequency of milk intake was associated with birth weight [16]. As dietary patterns using factor analysis have shown associations with birth size, this could perhaps be another useful method in this setting [6]. Thirdly, a minimum consumption amount of a food item was not required for it to be included in the DDS. Perhaps adding a cut-off value of e.g. 15 g, as suggested by Arimond et al., would have provided stronger associations as the DDS indicator would better reflect diet adequacy [1].

A major methodological strength of this study is the use of quantile regression, which enables a more comprehensive picture of the relationship between the independent and dependent variables. Whereas the ordinary least square method only provides the average effect of the independent variable on the dependent variable, quantile regression allows for the differential effect across the distribution of the dependent variables [5]. This could be helpful in detecting vulnerable subgroups and designing more appropriate interventions.

There are some limitations to this study. Around the time of the study, the proportion of Malawian women giving birth at health facilities was around 73%, although lower in rural than in urban areas [26]. To ensure follow-up at the health facilities we recruited only participants who intended to give birth at a health facility. Despite this, we only managed to collect data from 56.6% of the study participants. Home deliveries, identification challenges at health facilities and transfers to other facilities outside the study area, are likely reasons for this low follow-up rate. From focus group discussions with women in the area, several reasons for home deliveries were given, such as long distances to facilities, obligation to care for their older children, poor treatment at the facilities, and lack of transport money. This low follow-up rate could potentially introduce a bias in the results, but we found no differences in background characteristics or diet between those delivering at home and those delivering at health facilities. However, due to issues already mentioned, birth size could have been overestimated in our findings. Due to uncertain estimations of gestational age, some participants interviewed during one season could give birth in the following season. We categorized participants according to the season in which they gave birth. The participants’ DDS could possibly have changed between the time of the interview and time of birth (dietary interviews were conducted between 28 and 35 weeks of gestation). We tried to address this challenge by adjusting for the duration of food poor season (pre-harvest season) experienced by the participants. Moreover, there will be limitations when looking at post-harvest versus pre-harvest season, as there is no clear cut-point when one season starts and another ends. Another limitation of this study is that maternal dietary intake was only measured at one point during pregnancy. Previous studies have found that the associations between maternal intakes and birth outcomes differ throughout the pregnancy [27]. As fetal tissues and organs undergo different times of rapid development, the effect of diet on pregnancy outcomes will differ depending on when food shortages occur [28]. Maternal energy expenditure should ideally be adjusted for in the association analyses, however, we did not collect data on this.

## Conclusion

In conclusion, we found abdominal circumference and birth weight to be associated with seasonality in adjusted models. Our findings indicate a differential effect of seasonality across the distribution of birth size. We did not detect any statistically significant associations between DDS and birth size in the fully adjusted models. Larger studies should be conducted to investigate the effect of season and DDS on quantiles of birth size, as this method could help identify vulnerable subgroups and better target dietary interventions to improve birth outcomes.

## Supplementary Information

**Additional file 1: Table S1.** Association between birth seasons (pre-harvest vs post-harvest) and birth size.

## Data Availability

The datasets used and analyzed during the current study are available from the corresponding author on reasonable request.

## Abbreviations

CI: confidence interval
DDS: dietary diversity score
FAO: Food and Agriculture Organization of the United Nations
MDD-W: minimum dietary diversity score for women
TA: Traditional Authority

## References

[1] Arimond M, Wiesmann D, Becquey E, Carriquiry A, Daniels MC, Deitchler M (2010). Simple food group diversity indicators predict micronutrient adequacy of women's diets in 5 diverse, resource-poor settings. J Nutr.

[2] Food and Agriculture Organization. Minimum dietary diversity for women: a guide for measurement 2016. Internet: http://www.fao.org/3/a-i5486e.pdf. Accessed 13 Dec 2021.

[3] Kheirouri S, Alizadeh M. Maternal dietary diversity during pregnancy and risk of low birth weight in newborns: a systematic review. Public Health Nutr. 2021;24:4671–81. 10.1017/S1368980021000276 PMC10195329 33472725

[4] Saaka M (2012). Maternal dietary diversity and infant outcome of pregnant women in northern Ghana. Int J Child Health Nutr.

[5] Koenker R, Bassett G (1978). Regression quantiles. Econometrica..

[6] Mitku AA, Zewotir T, North D, Jeena P, Naidoo RN (2020). The differential effect of maternal dietary patterns on quantiles of Birthweight. BMC Public Health.

[7] UNICEF. Causes of malnutrition: Part 1: Fact sheet: UNICEF; 2011. Internet: http://www.unicef.org/nutritioncluster/files/Module5CausesOfMalnutritionFactSheet.pdf. Accessed 13 Dec 2021.

[8] Rao S, Kanade AN, Yajnik CS, Fall CH (2009). Seasonality in maternal intake and activity influence offspring's birth size among rural Indian mothers-Pune maternal nutrition study. Int J Epidemiol.

[9] Dominguez-Salas P, Moore SE, Baker MS, Bergen AW, Cox SE, Dyer RA (2014). Maternal nutrition at conception modulates DNA methylation of human metastable epialleles. Nat Commun.

[10] National Statistical Office. Malawi DHS 2010 - Final report 2011 Internet: https://dhsprogram.com/publications/index.cfm. Accessed 13 Dec 2021.

[11] National Statistics Office of Malawi UNPF. Malawi Population and Housing Census 2018 Internet: https://malawi.unfpa.org/. Accessed 13 Dec 2021.

[12] Harrigan J (2008). Food insecurity, poverty and the Malawi starter pack: false start or fresh start?. Food Policy.

[13] Kirkwood BR, Sterne JA. Essential medical statistics. 2nd ed. Oxford: Blackwell Publishing; 2003.

[14] Dean A, Sullivan K, Soe M. Open source epidemiologic statistics for public health; 2013.

[15] Gibson R, Ferguson E. An interactive 24-hour recall for assessing the adequacy of iron and zinc intakes in developing countries. Washington, D.C: Int Food Policy Res Inst; 2008.

[16] Hjertholm KG, Iversen PO, Holmboe-Ottesen G, Mdala I, Munthali A, Maleta K, et al. Maternal dietary intake during pregnancy and its association to birth size in rural Malawi: a cross-sectional study. Matern Child Nutr. 2018;14:e12433. 10.1111/mcn.12433 PMC6866046 28217860

[17] Hjertholm KG, Holmboe-Ottesen G, Iversen PO, Mdala I, Munthali A, Maleta K, et al. Seasonality in associations between dietary diversity scores and nutrient adequacy ratios among pregnant women in rural Malawi - a cross-sectional study. Food. Nutr Res. 2019;63. 10.29219/fnr.v63.2712 PMC6397333 30837821

[18] Food and Agriculture Organization (2012). Guidelines for measuring household and individual dietary diversity.

[19] Kamudoni P, Maleta K, Shi Z, Holmboe-Ottesen G (2007). Infant feeding practices in the first 6 months and associated factors in a rural and semiurban community in Mangochi District, Malawi. J Hum Lact.

[20] International Fetal and Newborn Growth Consortium (2012). The international fetal and newborn growth standards for the 21st century (INTERGROWTH-21ST): anthropometry handbook.

[21] de Onis M, Garza C, Victora CG, Onyango AW, Frongillo EA, Martines J (2004). The WHO multicentre growth reference study: planning, study design, and methodology. Food Nutr Bull.

[22] Hartikainen H, Maleta K, Kulmala T, Ashorn P (2005). Seasonality of gestational weight gain and foetal growth in rural Malawi. East Afr Med J.

[23] Johnson TS, Engstrom JL (2002). State of the science in measurement of infant size at birth. Newborn Infant Nurs Rev.

[24] Abubakari A, Jahn A (2016). Maternal dietary patterns and practices and birth weight in northern Ghana. PLoS One.

[25] Zerfu TA, Umeta M, Baye K (2016). Dietary diversity during pregnancy is associated with reduced risk of maternal anemia, preterm delivery, and low birth weight in a prospective cohort study in rural Ethiopia. Am J Clin Nutr.

[26] National Statistical Office - NSO/Malawi, ICF Macro (2011). Malawi demographic and health survey 2010.

[27] Rao S, Yajnik CS, Kanade A, Fall CH, Margetts BM, Jackson AA (2001). Intake of micronutrient-rich foods in rural Indian mothers is associated with the size of their babies at birth: Pune maternal nutrition study. J Nutr.

[28] Abu-Saad K, Fraser D (2010). Maternal nutrition and birth outcomes. Epidemiol Rev.

