# Supplementary material for: The impact of dietary diversity and seasonality in food availability on the quantile distribution of birth size among pregnant women in rural Malawi – a cross-sectional study
Source: BMC Pregnancy Childbirth. 2022 Jul 27;22:602. doi: 10.1186/s12884-022-04924-4 (PMC9327336; doi:10.1186/s12884-022-04924-4)
Supplement: Supplementary file 1 — Additional file 1: Table S1. Association between birth seasons (pre-harvest vs post-harvest) and birth size. [file 12884_2022_4924_MOESM1_ESM.pdf]

Additional File 1

Table S1 Association between birth seasons (pre-harvest vs post-harvest) and birth size

		25th quantile (95% CI)	50th quantile (95% CI)	70th quantile (95% CI)	90th quantile (95% CI)
**Birth weight**					
	Unadjusted	10<br>(-167.7 - 187.7)	-25<br>(-184.7 - 134.7)	-14<br>(-189.5 - 161.5)	350**<br>(129.9 - 570.1)
	Adjusted	-126.0<br>(-321.6 - 69.58)	-113.0<br>(-354.5 - 128.6)	-159.4<br>(-377.5 - 58.65)	320.2<br>(-2.882 - 643.3)
**Birth length**					
	Unadjusted	0.3<br>(-0.4 - 1.0)	-0.2<br>(-0.9 - 0.5)	-0.2<br>(-1.5 - 1.1)	0.5<br>(-1.2 - 2.2)
	Adjusted	0.2<br>(-0.6 - 1.0)	-0.4<br>(-1.0 - 0.3)	-0.4<br>(-1.5 - 0.7)	0.0<br>(-1.9 - 2.0)
**Head circumference**					
	Unadjusted	0.3<br>(-0.2 - 0.8)	0.1<br>(-0.6 - 0.8)	0<br>(-0.8 - 0.8)	-0.1<br>(-0.8 - 0.6)
	Adjusted	-0.2<br>(-1.2 - 0.9)	-0.4<br>(-1.8 - 1.1)	-0.2<br>(-1.2 - 0.8)	0.0<br>(-0.9 – 1.0)
**Abdominal circumference**					
	Unadjusted	0.6*<br>(0.1 - 1.1)	0.4<br>(0.0 - 0.8)	0.3<br>(-0.2 - 0.8)	-0.1<br>(-1.2 - 1.0)
	Adjusted	1.0**<br>(0.4 - 1.6)	0.5<br>(-0.4 - 1.4)	0.8<br>(-0.1 - 1.7)	0.4<br>(-1.5 - 2.4)

Data is presented as quantile regression estimates and 95% confidence intervals (CI) of birth seasons for the 25th, 50th, 70th and 90th quantiles of birth outcomes (n=190). Adjusted for maternal age, household assets, maternal education (yes vs no), birth season, interview season, total duration of food poor season during pregnancy and maternal energy intake. *P<0.05, **P<0.01.
